# Comparison of Receptive Verbal Abilities Assessed Using the KBIT-2 and BPVS3 in Adults With Down Syndrome

**DOI:** 10.3389/fpsyg.2018.02730

**Published:** 2019-01-17

**Authors:** Carla M. Startin, Sarah Hamburg, Andre Strydom, LonDownS Consortium

**Affiliations:** ^1^Department of Forensic and Neurodevelopmental Sciences, Institute of Psychiatry, Psychology and Neuroscience, King’s College London, London, United Kingdom; ^2^Division of Psychiatry, University College London, London, United Kingdom; ^3^LonDownS Consortium, London, United Kingdom

**Keywords:** Down syndrome, intellectual disability, receptive verbal ability, Kaufmann Brief Intelligence Test 2 (KBIT-2), British Picture Vocabulary Scale 3 (BPVS3)

## Abstract

Down syndrome (DS) is the most common genetic cause of intellectual disability. There is, however, considerable variation in cognitive abilities between those with DS, with some individuals scoring at floor on some tests, particularly for age-standardised outcomes. This variation and these floor effects can pose a problem for comparing and combining study populations when different standardised measures have been used to assess individuals’ cognitive abilities, for example combining results across studies to investigate genetic or other factors associated with cognitive abilities. To facilitate this comparison and combination of study populations assessed using different tests of verbal abilities, we administered two commonly used standardised tests of receptive language, the Kaufmann Brief Intelligence Test 2 (KBIT-2) verbal scale and the British Picture Vocabulary Scale 3 (BPVS3) to 34 adults with DS (age range 19–59) to investigate relationships between outcomes for these two tests. We found a very strong correlation between raw scores for the KBIT-2 verbal scale and the BPVS3, and determined equations to convert between scores for the two tests. Intraclass correlations between the two scales for age-equivalents and calculated *z* scores relative to population norms were also strong, though scores for both outcomes were significantly higher for the KBIT-2 verbal scale compared to the BPVS3. This deviation in scores between the two tests was greater as *z* scores decreased for both tests (i.e., for lower scoring individuals), with no such relationship observed for age-equivalents. These results indicate the conversion of raw scores between the KBIT-2 verbal scale and the BPVS3 may be a more valid method for the comparison or combination of study samples with DS compared to the use of standardised scores. Such comparisons or combinations will aid our understanding of cognitive variations and factors associated with these variations within the population with DS.

## Introduction

Down syndrome (DS), caused by the triplication of chromosome 21, is the most common genetic cause of intellectual disability (ID), and has a UK live birth incidence of approximately one in 1000 (Wu and Morris, 2013). Despite the presence of ID being almost universal in people with DS, with a mean IQ of 50, there is considerable variation in cognitive ability between individuals (Karmiloff-Smith et al., 2016; Startin et al., 2016). This variation, with some individuals performing at floor level on some tests, means it is important to be able to compare the cognitive abilities between study samples to understand how representative participants are and how generalizable study results may be. Further, several on-going studies aim to determine factors associated with differences in cognitive abilities between individuals with DS, including genetic, health, and demographic factors (Del Hoyo et al., 2016; Startin et al., 2016; Rosser et al., 2018). Determining such factors will require large sample sizes, in particular when considering genetic variations, and many current studies are therefore likely underpowered. To increase power, it will be important for studies to join datasets where possible.

To facilitate the comparison and combination of datasets from studies that have used different cognitive outcome measures it is vital to understand how scores on different tests compare. Ideal measures for comparison or combination will be from commonly used tests, with tests of IQ being one such likely candidate as these are typically included in studies as a measure of general cognitive abilities. Several IQ tests have previously been used in studies assessing cognitive abilities in adults with DS, including tests of both verbal and non-verbal abilities [see Hamburg et al. (unpublished) for a review]. The standardisation of IQ tests typically allows for combination of age-adjusted IQ scores between studies in individuals who do not have an ID, however, in studies of adults with DS high floor effects are observed when converting to standardised IQ scores (de Sola et al., 2015; Liogier d’Ardhuy et al., 2015; Startin et al., 2016). This is problematic for studies assessing differences in abilities, as high numbers of IQ scores at floor are not sensitive to these differences (Sansone et al., 2014). In addition, when comparing results from different IQ tests in adults with ID it has been reported standardised IQ scores are consistently higher for the Wechsler Adult Intelligence Scale (WAIS) compared to the Stanford–Binet Intelligence Scale (SBS; Silverman et al., 2010), therefore limiting the use of these measures for comparisons.

Age-equivalent scores may provide an alternative to standardised IQ scores, although floor effects are still possible, albeit to a smaller extent than for standardised IQ scores. Further issues with age-equivalent scores come from their interpretation. Firstly, these scores describe the age at which a particular raw score is average, and do not account for the variation in raw scores at that age. Secondly, interpreting the difference between two age-equivalent scores depends upon the scores themselves as development is not linear; for example differences of 6 months for ages 5 and 5.5 years and for ages 12 and 12.5 years are not equivalent (Mervis and Klein-Tasman, 2004; Maloney and Larrivee, 2007). In addition, both standardised IQ scores and age-equivalents assume typical development, which is not the case for individuals with an ID. Raw scores may therefore be the preferred outcome measure to use for studies assessing variability in cognitive abilities between adults with ID, though their use in children of different ages for this purpose may be limited.

Unlike IQ scores and age-equivalents, however, there is no standardisation for raw scores to allow for combination between different tests. To combine study results it is therefore essential to establish the relationship between raw scores for different tests. To aid in this, we administered two commonly used standardised tests of receptive language (the Kaufmann Brief Intelligence Test 2 (KBIT-2) verbal scale and the British Picture Vocabulary Scale 3 (BPVS3)) to a sample of individuals with DS to explore the relationship between scores for these tests. These tests were selected based on their easy and relatively quick administration requiring minimal verbal responses and previous use in a range of adults with DS, with test performance being considered a proxy for general cognitive abilities (Glenn and Cunningham, 2005; Strydom et al., 2009; de Sola et al., 2015; Startin et al., 2016). We also compared age-equivalent scores and generated *z* scores relative to population norms (which allowed us to assess deviation from typical performance) between the tests, to investigate the potential use of standardised measures to combine results from these two tests.

## Materials and Methods

### Participants

We recruited 34 adults aged 18+ years with DS (mean age 36.47 years, standard deviation 11.69, range 19–59) from the LonDownS Consortium adult participant pool (Startin et al., 2016). Participants included 17 males and 17 females, and from carer report based on everyday functional descriptions of the participants’ peak level of functioning adapted from the International Statistical Classification of Diseases and Related Health Problems Tenth Revision (ICD10), 16 (47.1%) individuals had a mild ID, 15 (44.1%) individuals had a moderate ID, and 3 (8.8%) individuals had a severe/profound ID. All adults had genetically confirmed triplication of chromosome 21, with trisomy 21 in 32 individuals, a translocation in one individual, and mosaic DS in one individual. Neither individual with non-trisomy 21 DS was an outlier in performance on the KBIT-2 or BPVS3. Several participants had comorbid psychiatric or neurological diagnoses; six with a history of epilepsy or seizures, two with autism, and one each with dementia, depression, anxiety, and bipolar disorder. One additional individual was originally recruited, but was unable to complete the first test due to vision problems and so was excluded from further testing.

Ethical approval was obtained for the LonDownS study from the North West Wales Research Ethics Committee (13/WA/0194). Where individuals had capacity to consent for themselves we obtained written informed consent in accordance with the Declaration of Helsinki. Where individuals did not have capacity to consent for themselves, a consultee was appointed and asked to sign a form to indicate their decision regarding the individuals’ inclusion based on their knowledge of the individual and his/her wishes, in accordance with the UK Mental Capacity Act 2005.

### Assessment

Participants were administered the KBIT-2 (Kaufman and Kaufman, 2004) and the BPVS3 (Dunn and Dunn, 2009); tests were administered in a counter-balanced order. Assessments took place in a testing room at University College London.

The KBIT-2 was designed to measure verbal and non-verbal intelligence of individuals aged 4–90 years, and consists of a verbal and non-verbal scale, with the verbal scale containing two subtests, assessing verbal knowledge and riddles. For the verbal knowledge subtest then participants are given a word and asked to select the correct picture corresponding to the word from six options, and for the riddles subtest then participants are asked to identify the item described in a short riddle. Each subtest was started at item 1 and stopped after four consecutive incorrect answers.

The BPVS3 was designed to measure receptive vocabulary for Standard English, particularly for individuals aged 3–16 years, but also being suitable for adults, and contains a verbal knowledge test. In this test participants are given a word and asked to select the correct picture corresponding to the word from four options. The starting point was chosen based on researchers’ prior knowledge of the individuals’ verbal ability; all individuals had participated in our previous studies (Startin et al., 2016), which included a previous KBIT-2 assessment performed at least two years prior to the current study. This time delay was adequate to prevent potential practise effects, and allowed us to estimate approximate verbal age-equivalents for the present study to determine the starting point for the BPVS3. For each individual the basal and ceiling sets were determined; these were the lowest set with 0 or 1 errors and the highest set with 8 or more errors, respectively.

Starting points for test administration for the KBIT-2 and BPVS3 were therefore adapted as appropriate from the manuals to be suitable for a population with ID; for both tests the manuals provide different starting points based on individuals’ chronological age.

### Statistical Analysis

All analysis was performed using SPSS version 22. Raw scores were first determined for the KBIT-2 verbal scale and BPVS3. Using standardised tables provided in the test manuals, scores were converted to age-equivalents, and to *z* scores relative to population norms. Sansone et al. (2014) suggested such *z* scores are a suitable alternative to standardised scores in those with an ID to determine deviations from typical age-expected performance across ages and test domains, due to the high floor effects when converting raw scores to standardised scores. *z* scores were calculated using the means and standard deviations in provided tables using the below formula:

$$z(i)=\frac{\left(score\;(i)-mean\;(j)\right)}{standard\;deviation\;(j)}$$

where individual *i* is in age group *j*. Using this formula, a *z* score of 0 represents typical age-expected performance, while a *z* score of –1 represents performance one standard deviation below this. For the BPVS3 the oldest age group with available means and standard deviations was 16 years 9 months to 16 years 11 months, and so the mean and standard deviation for this age group was used to determine *z* scores for all individuals as individuals in our sample were aged 19 years and above. For the KBIT-2 verbal scale, we therefore used the mean and standard deviation for a comparable age group to determine *z* scores for all individuals (equivalent norms in the KBIT-2 are for individuals aged 16 years 0 months to 16 years 11 months). The BPVS3 does not provide standardised IQ scores below 70, and so no comparisons were made for standardised IQ scores between the KBIT-2 verbal scale and BPVS3.

Firstly, we performed Pearson’s correlational analysis to determine the relationship between raw KBIT-2 verbal scores and BPVS3 scores. Based on this relationship we then determined linear regression equations to convert between test scores. Using these equations we calculated predicted KBIT-2 verbal scores and BPVS3 scores based on actual raw scores from the other test, and compared the absolute values of raw and predicted scores within each test using (a) intraclass correlational analysis and (b) paired samples *t*-tests. Intraclass correlational analysis assesses the reliability of scores by assessing absolute agreement, accounting for their absolute values in addition to the relationship between them.

Secondly, we performed (a) intraclass correlational analysis and (b) paired samples *t*-tests to compare absolute values of age-equivalents and *z* scores between KBIT-2 verbal scores and BPVS3 scores. Based on these results, we also performed Pearson’s correlational analysis for age-equivalents and *z* scores to determine whether deviation in scores between the two tests was associated with absolute scores on either test.

## Results

**FIGURE 1:**
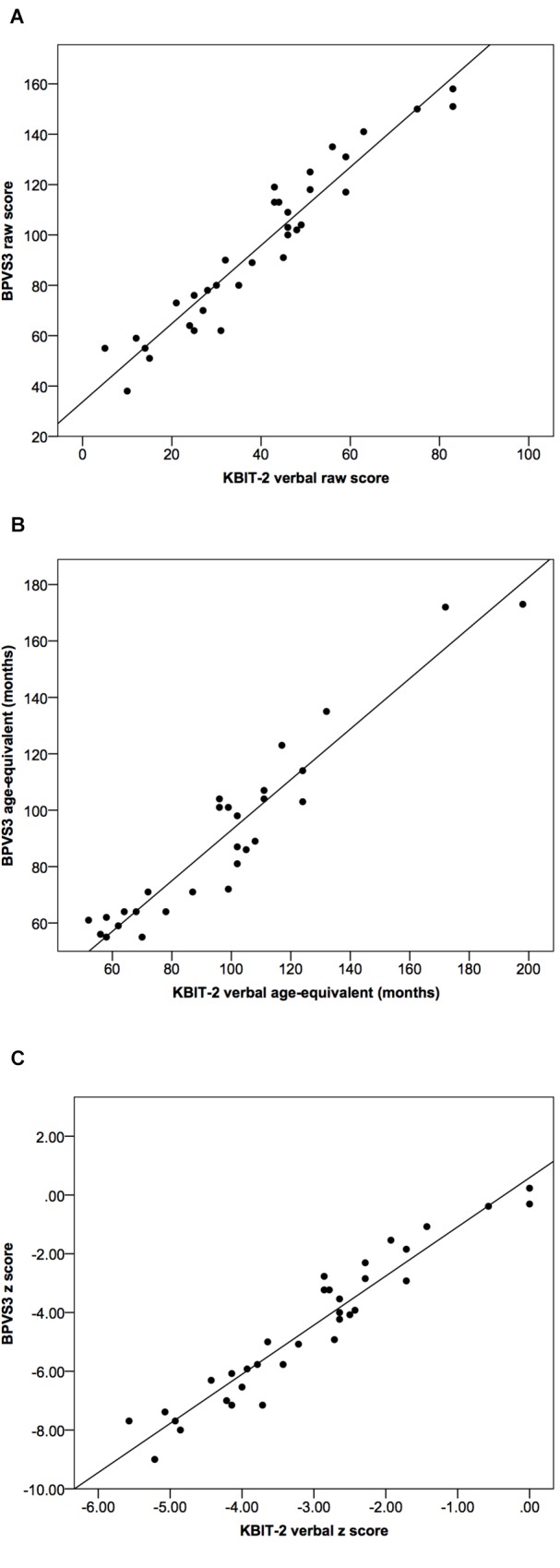
Scatter plots demonstrating relationships between KBIT-2 verbal scores and BPVS3 scores for **(A)** raw scores, **(B)** age-equivalents, and **(C)** *z* scores relative to population norms. Lines indicate least squares regression lines.

Information on raw scores, predicted scores, age-equivalents, and *z* scores relative to population norms for the KBIT-2 verbal scale and BPVS3 can be seen in Table 1 [full dataset available at Startin et al. (2018)]. Scatter plots demonstrating the relationships between raw scores, age-equivalents, and *z* scores for KBIT-2 verbal scores and BPVS3 scores can be seen in Figure 1. For age-equivalent scores, five individuals were at floor, and one individual was at ceiling for at least one test. These individuals were therefore not included in any analysis using age-equivalent values, leaving 28 individuals in the sample for such analysis. Converting raw scores to standardised IQ scores revealed high floor effects as expected, in particular for the BPVS3 which has a higher floor compared to the KBIT-2 verbal scale (scores of 70 vs. 40; *n* = 27 and *n* = 15 at floor, respectively).

**Table 1:** Raw scores, predicted scores, age-equivalents, and *z* scores relative to population norms for KBIT-2 verbal scores and BPVS3 scores.

	KBIT-2 verbal scores	BPVS3 scores
Raw score	40.06 ± 19.73 (5, 83)	95.94 ± 31.99 (38, 158)
Predicted score	39.98 ± 18.87 (5.79, 76.59)	95.81 ± 30.58 (41.47, 162.37)
Age-equivalent (months)^a^	97.25 ± 34.04 (52, 198)	90.43 ± 32.10 (55, 173)
*z* score	–3.07 ± 1.41 (–5.57, 0.00)	–4.54 ± 2.46 (–9.00, 0.23)

*Values given are mean ± standard deviation (minimum, maximum). ^a^Only participants with age-equivalents not at floor or ceiling included (n = 28).*

Correlational analysis indicated a very strong correlation between raw scores for the KBIT-2 verbal scale and the BPVS3 (*r* = 0.96, *p* > 0.001, see Figure 1); the linear regression equations to convert between scores for the two tests are given below:

$$\begin{array}{l} \text{BPVS}3\text{ score}=\text{(}1.55\times \text{KBIT-2 verbal score)}+33.72\\ \text{KBIT-2 verbal score}=\text{(}0.59\times \text{BPVS3 score)}-16.63 \end{array}$$

**FIGURE 2:**
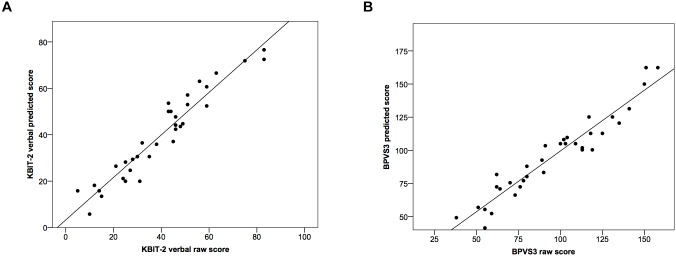
Scatter plots demonstrating relationships between raw scores and predicted scores for **(A)** the KBIT-2 verbal scale and **(B)** the BPVS3. Lines indicate least squares regression lines.

Using these formulae we calculated predicted BPVS3 scores based on actual raw KBIT-2 verbal scores, and predicted KBIT-2 verbal scores based on actual raw BPVS3 scores (Figure 2). Comparing raw and predicted KBIT-2 verbal scores indicated a high intraclass correlation coefficient between the scores (0.96, 95% CI (0.92, 0.98), *p* < 0.001) with no significant difference between the two scores [*t*(33) = 0.09, *p* = 0.932, mean difference = 0.08 ± 5.66, 95% CI (–1.89, 2.06)]. Similarly, comparing raw and predicted BPVS3 scores indicated a high intraclass correlation coefficient between the scores (0.96, 95% CI (0.92, 0.98), *p* < 0.001) with no significant difference between the two scores [*t*(33) = 0.08, *p* = 0.935, mean difference = 0.13 ± 9.17, 95% CI (–3.07, 3.33)].

Intraclass correlation coefficients for age-equivalents (Figure 1B) and *z* scores (Figure 1C) between the two tests were 0.93 (95% CI (0.79, 0.97), *p* < 0.001) and 0.65 (95% CI (–0.04, 0.88), *p* < 0.001), respectively.

Both age-equivalents and *z* scores were significantly higher for KBIT-2 verbal scores compared to BPVS3 scores [age-equivalents: *t*(27) = 3.42, *p* = 0.002, mean difference = 6.82 ± 10.56, 95% CI (2.73, 10.91); *z* scores: *t*(33) = 7.28, *p* < 0.001, mean difference = 1.48 ± 1.18, 95% CI (1.06, 1.89)].

Based on these results, which indicated differences in absolute values of age-equivalents and *z* scores between KBIT-2 verbal scores and BPVS3 scores, we finally determined whether there were any relationships between the size of these differences and the values of age-equivalents and *z* scores respectively for the KBIT-2 verbal scale and BPVS3, i.e., whether the degree of deviation in scores from the two tests was associated with scores on either test.

**FIGURE 3:**
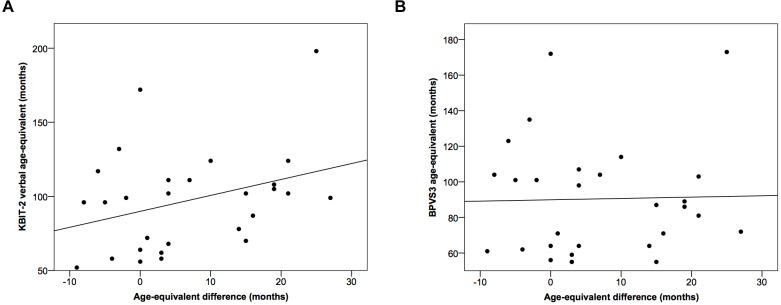
Scatter plots demonstrating relationships between the difference in age-equivalents for the two tests and age-equivalent scores using **(A)** the KBIT-2 verbal scale and **(B)** the BPVS3. Lines indicate least squares regression lines.

There was no significant correlational relationship between the difference in age-equivalents for the two tests and age-equivalent scores using either the KBIT-2 verbal scale (*r* = 0.33, *p* = 0.083) or the BPVS3 (*r* = 0.03, *p* = 0.901) (Figure 3).

**FIGURE 4:**
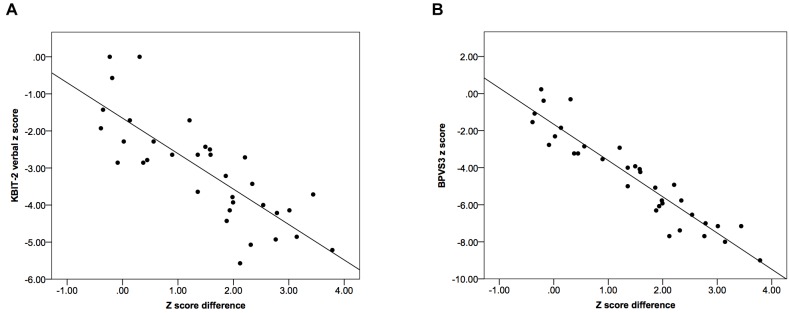
Scatter plots demonstrating relationships between the difference in *z* scores relative to population norms for the two tests and *z* scores using **(A)** the KBIT-2 verbal scale and **(B)** the BPVS3. Lines indicate least squares regression lines.

In comparison, there were significant strong negative correlational relationships between the difference in *z* scores for the two tests and *z* scores for both the KBIT-2 verbal scale (*r* = –0.80, *p* < 0.001) and the BPVS3 (*r* = –0.94, *p* < 0.001) (Figure 4).

These results indicated there was a greater deviation in *z* scores between the two tests as *z* scores decreased (i.e., for individuals with lower scores), while no such relationship was observed for age-equivalents.

## Discussion

We present results comparing scores from two commonly used tests to assess receptive verbal abilities in individuals with DS, the KBIT-2 verbal scale and the BPVS3, with scores on these tests considered as a proxy for general cognitive abilities. Raw scores showed a very strong correlation between tests, and we determined equations for converting scores between the tests. Using these equations to predict test scores, we demonstrated very high intraclass correlation coefficients with very small mean differences between actual raw scores and predicted scores for both the KBIT-2 verbal scale and the BPVS3, indicating the high reliability of the equations. These equations therefore allow for the reliable conversion of scores between the KBIT-2 verbal scale and the BPVS3, with the very strong correlation between scores for these two tests indicating they assess the same underlying construct(s). This conversion will allow for the comparison or combination of data from studies using these two tests, with the latter allowing for better-powered studies requiring large sample sizes, such as those investigating genetic associations.

In addition, we compared age-equivalents and calculated *z* scores relative to population norms for the KBIT-2 verbal scale and the BPVS3. Intraclass correlation coefficients between both age-equivalents and *z* scores for the two tests were high. However, scores for both outcomes were significantly higher for the KBIT-2 verbal scale compared to the BPVS3, with the mean values of these scores suggesting the BPVS3 to give an age-equivalent of approximately 6 months younger and a *z* score one and a half standard deviations further from typical performance compared to the KBIT-2 verbal scale. This indicates a systematic difference in these standardised scores, suggesting it may be unreliable to compare or combine them. Further, this deviation in *z* scores between the two tests was greater for those with poorer abilities, indicating the comparison of *z* scores from the two tests becomes more unreliable as abilities decrease, with no such relationship observed for age-equivalents. Though analysis for age-equivalents did not include those with an age-equivalent at floor or ceiling, excluding these individuals from analysis for *z* scores did not change the patterns of results observed (results not presented). Based on our results we are unable to determine whether the BPVS3 is underestimating scores or the KBIT-2 verbal scale is overestimating these, or a combination of the two. In addition, for the calculated *z* scores, this difference may suggest that the BPVS3 has a lower floor compared to the KBIT-2 verbal scale.

Our results therefore indicate age-equivalents and *z* scores are not suitable for use to compare or combine study samples and results using these two tests. As previously reported, high floor effects in age-adjusted standardised IQ scores for individuals with an ID means these scores are not sensitive to individual differences for those at floor, and so are also often not suitable for use (Sansone et al., 2014; Startin et al., 2016). Here, we also found several participants were at floor for age-equivalent scores, and one participant was at ceiling. Our equations for the conversion of raw scores between the KBIT-2 verbal scale and BPVS3 therefore offer a suitable alternative for comparing or combining data that is more valid than using age-equivalents or *z* scores.

Our finding of higher age-equivalents and *z* scores relative to population norms for the KBIT-2 verbal scale compared to the BPVS3 also indicates it may be unreliable to use these scores to assess individuals’ abilities relative to population norms. However, these scores may be useful to give a rough approximation compared to age-typical levels and to track individuals’ development when the same test is administered over time. Alternatively, growth scores (which assess change within an individual and do not consider norm values) are often useful for tracking change within individuals with ID.

In addition to understanding how scores on different tests relate to each other being essential to compare or combine datasets, it is important to understand these relationships when IQ tests may be used to assess individuals to determine their needs and appropriate support. Silverman et al. (2010) compared standardised scores from the WAIS and SBS in a sample of adults with ID, finding large differences in IQ for the two tests. As such results may impact upon the assessed needs of individuals it is important to ensure results using different tests are reliable. Related to this, IQ scores may be less reliable in those with an ID due to such individuals not being represented in standardisation samples and poor test sensitivity for individuals performing at the lower end of the spectrum with high floor effects. Developing a reliable method to assess individuals’ abilities is therefore essential for research and clinical service provision in populations with an ID.

The main limitation of our study relates to the use of population data to determine age-equivalents and *z* scores, with population data obtained from the KBIT-2 and BPVS3 manuals. Firstly, the population data available was not collected from individuals with DS, which may limit its generalizability for converted age-equivalents and *z* scores in the present study. Secondly, differences within the populations used to obtain population data may impact upon its utility; KBIT-2 population data was obtained from individuals in the United States, and BPVS3 population data was obtained from individuals in the United Kingdom. Finally, based on the availability of population data from the BPVS3 we used the population mean and standard deviation for individuals aged 16 years 9 months to 16 years 11 months to calculate *z* scores (the oldest available age group). Although the BPVS3 is suitable for use in adults, it is typically used in individuals aged 16 years and under, and norms are only available for individuals aged 3 to 16 years. To improve comparisons we used population data from a similar age group to calculate *z* scores for the KBIT-2 verbal scale (individuals aged 16 years 0 months to 16 years 11 months). The use of population data from these age groups rather than using population data for adults with a similar chronological age to participants may therefore have altered individuals’ “true” *z* scores relative to population norms. As our data analysis was comparing *z* scores for the two tests rather than interpreting *z* scores relative to population norms this does not affect our conclusions, but is an important consideration to note. Another limitation relates to our sample size; while this was sufficient to demonstrate a number of significant relationships in our data, a larger sample may aid in confirming possible relationships between the difference in age-equivalents for the two tests and age-equivalent scores using either the KBIT-2 verbal scale or the BPVS3.

Within this study, participants demonstrated a range of scores on the KBIT-2 verbal scale and BPVS3, suggesting the results presented here should be relevant to a wide range of studies of individuals with DS. We recruited individuals with a wide age range, with non-trisomy 21 DS, and with psychiatric and neurological comorbidities to ensure results were generalizable to the wide spectrum of individuals with DS. As analysis compared results within individuals rather than between individuals such variations do not impact upon the reliability of our conclusions. Future studies should also include other populations with an ID or younger individuals with DS to determine whether these results may be applicable to these other populations, and also use other IQ tests (assessing both verbal and non-verbal abilities) to determine relationships between scores on different tests. Understanding these relationships will facilitate the comparison of studies, and allow for the combination of data from different studies, an essential first step in understanding population variations and factors associated with these.

## Members of the Londowns Consortium

The LonDownS Consortium principal investigators are Andre Strydom (chief investigator), Department of Forensic and Neurodevelopmental Sciences, Institute of Psychiatry, Psychology and Neuroscience, King’s College London, London, United Kingdom, and Division of Psychiatry, University College London, London, United Kingdom; Elizabeth Fisher, Department of Neurodegenerative Disease, Institute of Neurology, University College London, London, United Kingdom; Dean Nizetic, Blizard Institute, Barts and the London School of Medicine, Queen Mary University of London, London, United Kingdom, and Lee Kong Chian School of Medicine, Nanyang Technological University, Singapore, Singapore; John Hardy, Reta Lila Weston Institute, Institute of Neurology, University College London, London, United Kingdom, and United Kingdom Dementia Research Institute at UCL, London, United Kingdom; Victor Tybulewicz, Francis Crick Institute, London, United Kingdom, and Department of Medicine, Imperial College London, London, United Kingdom; and Annette Karmiloff-Smith, Birkbeck University of London, London, United Kingdom (deceased).

## Data Availability Statement

The dataset analysed for this study can be found in the figshare repository at https://doi.org/10.6084/m9.figshare.6553514.v2.

## Author Contributions

AS conceived the adult cohort study in conjunction with LonDownS principal investigators. CS designed the study and data analysis, analysed the data, and drafted the initial version of the report. CS and SH performed data collection. All authors contributed to revision and editing of the report.

## Conflict of Interest Statement

The authors declare that the research was conducted in the absence of any commercial or financial relationships that could be construed as a potential conflict of interest.
